# Retraction Note to: Diagnosis, classification and grading of canine mammary tumours as a model to study human breast cancer: an Clinico-Cytohistopathological study with environmental factors influencing public health and medicine

**DOI:** 10.1186/s12935-016-0358-6

**Published:** 2016-11-04

**Authors:** Radmehr Shafiee, Javad Javanbakh, Nahid Atyabi, Pegah Kheradmand, Danial Kheradmand, Alimohammad Bahrami, Hasti Daraei, Farshid Khadivar

**Affiliations:** 1 Faculty of Veterinary Medicine, Tehran University, Tehran, Iran; 2 Department of Pathology, Faculty of Veterinary Medicine, Tehran University, Tehran, Iran; 3 Semnan University of Medical Science, Faculty of Medicine, Semnan, Iran; 4 Graduate Islamic Azad University of Mashhad, Faculty of Medicine, Mashhad, Iran; 5 Graduate Paraveterinary Faculty of Ilam, University of Ilam, Ilam, Iran; 6 Department of Environmental Health Engineering, Alborz University of Medical Sciences, Karaj, Iran

## Retraction Note to: Cancer Cell International 2013, 13:79 DOI 10.1186/1475-2867-13-79

The Editors-in-Chief and Publisher have retracted this article [1] because the scientific integrity of the content cannot be guaranteed. An investigation by the Publisher found it to be one of a group of articles we have identified as showing evidence suggestive of attempts to subvert the peer review and publication system to inappropriately obtain or allocate authorship. This article showed evidence of plagiarism (most notably from the articles cited [2, 3]) and peer review and authorship manipulation.
